# Relationship of post-traumatic stress disorder with disrespect and abuse during childbirth in a group of Iranian postpartum women: a prospective study

**DOI:** 10.1186/s12991-021-00331-9

**Published:** 2021-01-23

**Authors:** Khadije Hajizadeh, Mojgan Mirghafourvand

**Affiliations:** 1 Midwifery Department, Tabriz University of Medical Sciences, Tabriz, Iran; 2 Social Determinants of Health Research Center, Tabriz University of Medical Sciences, Tabriz, Iran

**Keywords:** Disrespect, Abuse, Post-traumatic stress disorder, Longitudinal study, Iran

## Abstract

**Background:**

Disrespect and abuse (D&A) violate the fundamental principles of ethics, human rights, and basic obligations to protect and relieve patients. This study aimed to identify the status of D&A and its relationship with the post-traumatic stress disorder (PTSD) among Iranian women.

**Methods:**

This prospective study was conducted on 288 mothers admitted to the maternity wards of three public and three private hospitals in Tabriz. The data collection tools were socio-demographic and obstetric questionnaires as well as D&A (6–18 h postpartum) and PTSD (one month postpartum) scales, which were completed by participants in interviews. Multivariate logistic regression was employed to determine the relationship between PTSD and D&A in adjusting the socio-demographic and obstetric variables.

**Results:**

In this study, 74.7% of mothers reported one or multiple types of D&A. According to the diagnostic criteria for PTSD, 16.3% of the participants experienced postpartum PTSD. The mean (± standard deviation) of the PTSD score was reported 7.32 (± 2.0) in the women experiencing D&A, whereas it was 1.0 (± 0.0) in the women having no experiences of D&A. According to the Mann–Whitney U test results, there was a significant relationship between the total and all subscales of PTSD score and D&A (*p* < 0.001). The multivariate logistic regression results indicated that the likelihood of PTSD was significantly lower in the participants without any D&A experiences than in those with D&A experiences (aOR: 0.06; 95% CI 0.01 to 0.58; *p* = 0.015).

**Conclusion:**

Given the PTSD–D&A relationship, it is recommended to improve maternal care in maternity facilities to prevent any unintended PTSD complications.

## Background

The World Health Organization (WHO) published certain recommendations on attention to adequate and proper intrapartum care for positive childbirth experiences in 2018 by emphasizing the importance of respectful maternity care (RMC) [1]. Unfortunately, most women experience disrespect and abuse (D&A) instead of empathy, support, and respect in delivery rooms [2, 3]. In different studies, the prevalence of D&A varies from 15 to 98%. [4, 5]. Health workers’ negative behavior towards women in the delivery room increases fear of vaginal birth and C-section [3]. D&A can also cause long-term injuries and emotional traumas [6]. Since women are more physiologically, socially, and psychologically vulnerable during labor and delivery [7–9], D&A could exacerbate maternal psychological problems including sleep difficulty and the post-traumatic stress disorder (PTSD) [10].

In various studies, childbirth is considered a traumatic event in which 1.5–6% of women experience postpartum PTSD [11–13]. Birth traumas include traumatic experiences which are likely to occur at any stage of labor and may last up to a year afterwards. Postpartum psychological traumas often occur for a variety of reasons such as psychological distress (fear) during labor and delivery, obstetric complications (dystocia and emergency C-section) or the negative outcomes of pregnancy and childbirth (stillbirth and infant death). These traumas can result from physical or mental injuries following childbirth [14–17]. Some outcomes of postpartum PTSD include changes in mood and behavior (sadness, insensitivity, and depression) and alienation, sleep difficulty, isolation, loss of life expectancy, interference with women's social functions, negative impacts on matrimonial relationships (sexual dysfunction and disagreement), reduced emotional mother–baby connections, baby’s impaired psychological development, and experience of traumatic delivery [18, 19].

Beck et al*.* [20] identified the following factors as the causes of psychological trauma: lack of adequate and respectful maternity care (RMC), lack of proper communication between healthcare providers or service providers and mothers, lack of power and control, inattention to women's feelings, lack of privacy, inability to instill a sense of security in women, lack of empathy from care providers, and lack of sufficient information. Deprivation of RMC plays a major role in the traumatic childbirth experience, inasmuch as inadequate care makes mothers feel that they are forced to be in the delivery bed, exposed, and left with no help. Women perceive such care with fear and despair. They also experience a lack of proper communication from caregivers during labor and childbirth as a feeling of loneliness, fear, lack of support, and violence along with insecurity [20, 21].

The prevalence of PTSD and its relevant factors has been analyzed in many countries [11–13] including Iran [22, 23]; however, no studies have been conducted to quantitatively examine the relationship between D&A and PTSD. Given the high prevalence of postpartum PTSD and its negative outcomes as well as the importance of providing RMC and its potential role PTSD, this study aimed to analyze the relationship between PTSD and D&A during childbirth in the women admitted to the public and private hospitals of Tabriz.

## Methods

### Study design

This prospective study was conducted on 288 postpartum women admitted to the public and private hospitals of Tabriz (from October 7 to December 22, 2019).

### Statistical population

The eligibility criteria were for women to have experienced vaginal delivery in Tabriz and no self-reported history of depression or mental problems before or during pregnancy, taken no maternal antidepressants, experienced no stressful events such as divorce, death of the first-degree family members, and diagnosis of an incurable or refractory disease for a family member in the last 3 months, and had no major anomalies in the infant and infant death as well as lack of mental disability, deafness, and dumbness. The eligibility criteria were based on the participant self-reports.

### Data collection tools

The socio-demographic and obstetric details were collected through a researcher-made questionnaire, the face and content validities of which were confirmed. For evaluation, the questionnaire was distributed to 10 faculty members of Tabriz University of Medical Science. Based on their feedback, the necessary changes were made in consultation with the research team. The questionnaire consisted of four main sections, i.e., (1) socio-demographic details (mother's age, education, job, income status, and life satisfaction as well as husband's age, education and job); (2) obstetric details (history of abortion and number of pregnancies); (3) labor (time and duration of labor, birth attendant, gestational age at delivery, number of health care providers, type of hospital, augmentation, episiotomy, use of analgesics, postpartum hemorrhage, and prolonged labor), and (4) neonatal details (neonatal sex, hospitalization at NICU and Apgar score of 5 min, birth weight).

The D&A scale consists of 7 statements and 23 items including protecting women from physical harm (6 items), protecting their rights for acquiring information about their condition/informed consent/preferences (8 items), maintaining their confidentiality and privacy (1 item), maintaining their dignity and respect (2 items), providing them with equitable and discrimination-free care (2 items), taking care of pregnant women (never left without care/attention) (3 items), and discharging them upon their requests (never detaining or confining them against their will) (1 item). If any of the items in a statement are positive, then a D&A score will be considered for that statement. The scale was completed 6 to 18 h postpartum. It was designed by Asefa et al*.* in 2015 [24] and approved by the Maternal and Child Health Integrated Program (MCHIP) [25]. Validity and reliability of this questionnaire were assessed by the research team in another project, the paper of which is under review now. In the present study, Cronbach's alpha was reported 0.90, whereas ICC (with 95% CI) was obtained 0.98 (0.96 to 0.99).

The PTSD Symptom Scale (PSS-I) consists of 17 items that exactly cover all the criteria for the Diagnostic and Statistical Manual of Mental Disorders, Fourth Edition (DSM-IV) employed to diagnose the post-traumatic stress disorder. Moreover, the Likert scale was utilized to score the severity of symptoms in each criterion. The PTSD symptoms include the symptoms related to re-experiencing (4 items), avoidance (7 items), and motivational reactions (6 items). The PTSD was diagnosed in case of one or more re-experiencing related symptoms, three or more symptoms of avoidance, and two or more symptoms of motivational reactions [19]. The scores ranged from 0 to 51. Cronbach's alpha of the Persian version of the questionnaire was reported 0.88, whereas the kappa coefficient was measured 1 through the test–retest method [26].

### Sampling

After the ethical approval code (IR.TBZMED.REC.1398.1037) was obtained from the Ethics Committee of Tabriz University of Medical Sciences, the sampling process was performed in the maternity wards of public hospitals (Alzahra, Taleghani, 29 Bahman) and private hospitals (Behboud, Noor Nejat, Shahryar) of Tabriz. It was performed from October 7 to December 22, 2019. The study was conducted on 288 postpartum women based on the childbirth statistics in the three recent months prior to their participation. The author visited the hospitals, and all the women who gave birth in each hospital were evaluated for eligibility until the sample size was completed in that hospital. If eligible, the research objectives and methods were fully explained to them. If they wished to participate in the study, written informed consent was obtained from them. The socio-demographic and obstetric questionnaires and the D&A scale were completed 6 to 18 h postpartum with face-to-face interviews in private rooms at hospitals, whereas the PTSD scale was completed 1 month postpartum via phone calls. In the sampling process, whenever it was observed that participants suffered from PTSD based on the research checklist, they were immediately referred to a psychiatrist. In this study, 343 women were screened in terms of eligibility criteria, 16 women were excluded due to (2: history of depression, 2: death of first-degree family members, 1: had major anomalies in the infant, 3: infant’s death, 1: deafness). Moreover, 334 women were eligible for participation, whereas 288 women agreed to participate in this study.

### Sample size

A sample size of 120 was obtained from the results of a study in Ethiopia [24] about prevalence of D&A (*p* = 0.32), *q* = 0.68, *d* = 0.1 and and *Z* = 1.96. Considering the implementation of a study in public (*n* = 120) and private (*n* = 120) hospitals in Tabriz, 288 women were selected as the final sample by considering a 20% attrition rate.

### Data analysis

Data analysis was performed in SPSS 24. Descriptive statistics including frequency (%) and mean (standard deviation) were utilized to explain the socio-demographic and obstetric variables and the D&A scale. The Mann–Whitney *U* test was then conducted in the bivariate analysis to determine the relationship between D&A and PTSD scores due to the abnormal distribution of total PTSD and its subscales scores. The relationship of PTSD with the socio-demographic and obstetric characteristics was assessed first by using univariate logistic regression to calculate crude odds ratios (cORs). Variables with *p*-values < 0.2 were entered into backward multivariate logistic regression to control confounding. Results of multivariate logistic regression were presented as adjusted odds ratios (aOR) with a 95% confidence interval (CI). The significance level was considered *p* < 0.05.

## Results

### Participants' characteristics

Half of the participants (50.0%) were aged between 26 and 35 years old. Most of them (95.8%) were housewives. Nearly half of the participants (44.4%) had high school education and experienced the first childbirth (43.1%). In more than three-fourths of the women (78.1%), the gestational age was 37 weeks and more. In nearly half of the participants (43.7%), the hospital stay was less than 5 h, whereas in slightly more than half of the participants, delivery was performed during the day (56.6%) by a resident or on-call obstetric specialist (56.6%). Table 1 presents other details.

**Table 1:** Socio-demographic, pregnancy characteristics among participants (*n* = 288)

Variables	*n* %	Variables	*n* %	Variables	*n* %
Age (Years)		Marital satisfaction	214 (64.1)	Gravid	
18–25	12 (38.9)	Planned pregnancy	195 (67.7)	One	128 (43.1)
26–35	144 (50.0)	Husband’s education		Two	98 (34.0)
≥ 36	32 (11.1)	Elementary and lower	65 (19.5)	Three	47 (16.3)
Work status		Intermediate	72 (21.6)	≥ 4	19 (6.6)
House keeper	276 (95.8)	High school	132 (39.5)	Hospitalized in labour and delivery room (Hour)	
Employed	12 (4.2)	University	65 (19.5)	≤ 5	120 (41.7)
Infertility	8 (2.8)	Husband’s job		6–10	97 (33.7)
Prenatal class	65 (22.6)	Unemployed	12 (4.2)	≥ 11	71 (24.7)
Education		Employed	18 (6.3)	Birth attendant	
Elementary and lower	45 (15.6)	Self-employed	99 (34.4)	Midwife	71 (24.7)
Intermediate	66 (22.9)	Manual worker	159 (55.2)	Obstetrician (resident or on call)	163 (56.6)
High school	128 (44.4)	Abortion history		Student (midwifery or intern)	14 (4.9)
University	49 (17.0)	No abortion	224 (77.8)	Personal physician or midwife	40 (13.9)
Husband’s age (Years)		One	52 (18.1)	Number of health care provider	
18–25	31 (10.8)	≥ 2	12 (4.2)	One	41 (14.2)
26–35	172 (59.7)	Marital satisfaction		Two	149 (51.7)
≥ 36	85 (29.5)	Yes	195 (67.7)	≥ 3	98 (34.0)
Giving birth at day (8 a.m. to 8 p.m.)	163 (56.6)	No	93 (32.3)	Admission to NICU	39 (13.5)
Prolonged labour (dilatation < 1 cm/h)	33 (11.5)	Weight of baby (gram)		Augmentation	163 (56.6)
Use of the pain relief	88 (30.6)	≤ 2500	34 (11.8)	Doula presence	8 (2.8)
Gestational age at birth		2500–4000	243 (84.8)	APGAR at 5 min after birth	
< 37	63 (21.9)	≥ 4000	11 (3.8)	≤ 8	6 (2.1)
≥ 37	225 (78.1)	Baby sex (girl)	142 (49.3)	9–10	282 (97.9)
Postpartum bleeding	23 (8.0)	Episiotomy	236 (81.9)		

### Status of PTSD and D&A

Furthermore, 74.7% of the participants reported one or more types of D&A. Status of disrespect and abuse in the seven statements of D&A were as follows: physical harm or ill treatment (63.2%), woman’s right to information, informed consent (60.8%), confidentiality and privacy (31.3%), dignity and respect (16.7%), discrimination (7.6%), no attention (16.3%), and being detained or confined (1.0%).

The mean (± SD) PTSD total score was 5.72 (± 10.01) within the 0–51 range, whereas it was 1.90 (± 2.97) for re-experiencing within the 0–12 range or 2.00 (± 4.24) for avoidance within the 0–21 range; however, it was 1.80 (± 0.13) for arousal reaction within the 0–18 range (Table 2).

**Table 2:** The status of the PTSD and D&A in participants (*n* = 288)

Variable	Mean (SD)	Median (per 25 to 75)	Obtainable score range	Obtained score range
Total score of PTSD	5.72 (10.01)	1 (0–7)	0–51	0–51
Re-experiencing symptoms	1.90 (2.97)	0 (0–3)	0–12	0–12
Avoidance symptoms	2.00 (4.24)	0 (0–2)	0–21	0–21
Arousal symptoms	1.80 (0.13)	0 (0–2)	0–18	0–18
D&A	215 (74.7)^a^			

^a^Number (Percent)

According to the definition of PTSD (in case of having one or more symptoms related to re-experiencing, three or more symptoms related to avoidance, and two or more symptoms related to arousal reactions), 47 women (16.3%) experienced postpartum PTSD.

### Relationships of D&A and other significant factors with PTSD

The mean (± standard deviation) of the total PTSD score in women with D&A experience was 7.32 (± 2.0) and 1.0 (± 0.0) in women without D&A experience. There was a significant relationship between D&A and its subscales with overall PTSD scores based on the Mann–Whitney *U* test (*p* < 0.001) (Table 3).

**Table 3:** The relationship between PTSD and subscales with D&A (*n* = 288)

Variable	Women experiencing D&A Mean (SD)	Women without experiencing D&A Mean (SD)	*p*-value^a^
Total score of PTSD	7.32 (2.0)	1.0 (0.0)	< 0.001
Re-experiencing symptoms	2.41 (1.0)	0.4 (0.0)	< 0.001
Avoidance symptoms	2.60 (0.0)	0.2 (0.0)	< 0.001
Arousal symptoms	2.30 (0.0)	0.3 (0.0)	< 0.001

^**a**^Mann–Whitney test

There was a significant relationship between D&A with socio-demographic factors (marital satisfaction, number of pregnancies), intrapartum factors (time of birth, birth attendant, hospitalization duration in labor and delivery room, number of healthcare providers, augmentation with oxytocin, gestational at birth and source of support) and neonatal factors (baby’s gender) (*p* < 0.05).

Variables of marital satisfaction, hospitalization duration in labor and delivery room, number of healthcare providers, sources of support, and number of pregnancies were removed from the model. Multivariate logistic regression showed that not experiencing of D&A (aOR 0.065; 95% CI 0.007 to 0.58) and daytime childbirth (aOR 0.040; 95% CI 0.17 to 0.90) decreased the likelihood of PTSD. However, augmentation in labor rooms (aOR 5.15; 95% CI 2.29 to 11.56) and the attendance of student (intern or midwives) (aOR 15.71; 95% CI 1.99 to 123.72) increased the likelihood of PTSD (Table 4).

**Table 4:** Multivariable regression logistic model for PTSD and influencing factors (n = 288)

Variables	aOR (95% CI)^a^	*p*
D&A		
Experience of D&A (Ref)	1	
Not experiencing of D&A	0.06 (0.01 to 0.58)	0.015
Gestational age at birth		
> 37 (Ref)	1	
≤ 37	2.27 (1.00 to 5.18)	0.050
Augmentation		
No (Ref)	1	
Yes	5.15 (2.29 to 11.56)	< 0.001
Baby sex		
Boy (Ref)	1	
Girl	0.53 (0.250 to 1.12)	0.98
Time of giving birth		
Night (Ref)	1	
Day (8 a.m. to 8 p.m.)	0.40 (0.17 to 0.90)	0.027
Birth attendant		
Personal physician or midwife (Ref)	1	
Midwife	0.20 (0.02 to 1.77)	0.150
Obstetric (resident or on call)	1.60 (0.30 to 8.45)	0.576
Student (midwifery or intern)	15.71 (1.99 to 123.72)	0.009

Adjusted for all other socio-demographic variables with *p* < 0.2 in the bivariate analysis. Variable of marital satisfaction were removed from the model

Adjusted for all other pregnancy variables with *p* < 0.2 in the bivariate analysis. Variable of number of pregnancy was removed from the model

Adjusted for all labour and childbirth variables with *p* < 0.2 in the bivariate analysis. Variables of hospitalization duration in labour and delivery room, number of healthcare providers, source of support were removed from the model

^a^Adjusted odds ratio (95% confidence interval)

### Discussion

This study analyzed the PTSD status and its relationship with D&A in women giving birth at public and private hospitals of Tabriz. A high rate of D&A (74.7%) was reported by women. According to the diagnostic criteria for PTSD, 47 participants (16.3%) experienced postpartum PTSD. The highest PTSD score was related to avoidance, whereas the lowest score came from arousal reactions. The results showed that PTSD was significantly lower in participants without D&A experience than in those with D&A experience.

### Status of D&A

The research findings indicated a high rate of D&A (74.7%) in a group of Iranian women lower than D&A in other countries such as Nigeria (98%) [5] and Ethiopia (78%) [24] and higher than those of such countries as Kenya (20%) [3], Tanzania (15%) [27], India (57%) [28] and the European countries (20%) [29]. Compared to other nations such as European countries, a higher D&A score in Iran is observed apparently because most deliveries performed in Iran are not physiologic. In fact, they are induced by medical interventions. For instance, episiotomy and the augmentation during labor are considered routine and acceptable procedures in Iran [30, 31]. Higher subscales of D&A scores were physical harm or ill treatment statement (63.2%). This statement included physical violence, restraining, depriving of food or her baby without any medical permission and not receiving any painkiller [24]. According to the WHO, women are being mistreated during childbirth in health facilities all over the world. However, all women have the right to dignified, respectful health care and freedom [32, 33].

### Status of PTSD

According to the diagnostic criteria for PTSD, 47 participants (16.3%) experienced postpartum PTSD. In Iranian studies, a PTSD of 17–54% was reported [22, 23]. Compared to the global statistics (1.5–6%) [11–13], this is a high estimate which could be due to many interventions during pregnancy and childbirth in Iran or the cultural differences and women’s different expectations of the care received or perceived during the procedure.

### Relationship between D&A and PTSD

According to the results, PTSD was significantly lower in participants without any D&A experiences than in those with D&A experiences. In this regard, there are no studies on the quantitative assessment of the interplay between these categories. Findings of several studies indicated that healthcare providers' behavior during labor and delivery affected postpartum PTSD. Gurber et al*.* [34] studied 219 women in 1 month postpartum and indicated that support, care, and positive childbirth experience reduced acute stress and symptoms of postpartum depression. Boorman analyzed 890 Australian women (2009), 29% of whom felt threatened during labor, whereas 14% of them experienced loneliness and lack of support or panic. In that study, most women overcame the debilitating pain, fear, and anxiety of labor and delivery. However, in some women, the childbirth trauma remained deep and prolonged [35]. In a study conducted in Australia (2000), 499 women were interviewed in 4–6 weeks postpartum. The results indicated that women who reported high levels of obstetric interventions or dissatisfaction with the provided care were more likely to develop symptoms of mental trauma than the women who received adequate care [36]. In addition, staff's actions and interactions can influence PTSD after childbirth. In a qualitative study by Reed et al. [37] interviewing 748 women about relationship of PTSD with staff’s interactions, these themes were identified: threat, violence, unnecessary intervention, and prioritizing agenda over the needs of the women. Therefore, proper strategies should be adopted to reduce invasive procedures, prepare mothers with sufficient information about the possibility of unexpected events or patients’ decisions on emergency C-section and other interventions during delivery. Since some symptoms of postpartum trauma may have a delayed onset, emotional support and ongoing postpartum evaluation are essential.

### Relationship of other intrapartum significant factors and PTSD

Our results showed that daytime childbirth reduced the probability of PTSD. Furthermore, nighttime childbirth can increase the likelihood of tiredness and sleeping among care providers and physicians, something which may affect medical staff's behavior including aggression, anxiety, and depression and may ultimately cause disrespectful maternity care. Such behavior may worsen PTSD after childbirth with impacts on maternal fear, depression, and anxiety [38, 39].

The attendance of students (intern or midwives) increased the PTSD. This might be due to an anxious aspects of insecure attachment style. Availability, support and intimacy were violated in inappropriate attachments between care providers and woman, something which is a risk factor for women's major depression [40, 41].

Our results demonstrated that augmentation increased the chance of PTSD. Augmentation with oxytocin was proven to increase the probability of negative childbirth experience [42, 43]. Moreover, negative childbirth experience is related to PTSD after childbirth [44].

### Advantages and limitations

The inclusion of multiparous and primiparous women with vaginal delivery, term, and preterm as well as singleton and twin births can be considered the advantages of this study. Another advantage is the combined sampling of public and private hospitals. Moreover, none of the participants left the study. Attrition bias was minimized through accurate follow-up by phone contacts (twice a week).

However, a research limitation is that all participants were confined to Tabriz, and they were Azeri speakers in almost all cases. It is recommended to conduct a similar study in other (urban or rural) regions of Iran with different cultures and ethnicities. Another limitation is that psychometric properties of D&A scale were measured in Iranian postpartum women; however, this information has not been published yet. Since the D&A questionnaire was completed in the hospital setting, mothers may have under-reported D&A for the fear of not being able to use medical services after childbirth. Thus, it is recommended that future follow-up studies assess D&A after discharge from the hospital.

## Conclusion

This study showed high levels of D&A in childbirth, something which could be a major stressor for mothers. The research findings also showed that PTSD was significantly lower in participants without any D&A experiences than in those with D&A experiences. Hence, disrespectful and abusive care can be associated with PTSD. Given the importance of labor and delivery as well as the relationship between D&A and PTSD, policymakers must promote the normal-vaginal delivery (NVD) in the light of recognizing the status of D&A, protecting women from any disrespect of abuse, informing women about their rights, focusing on the importance of empathy, and adopting specific strategies to mitigate disrespect and abuse in the delivery room. Service providers should pay more attention to women during labor and childbirth.

## Data Availability

The datasets used and analyzed in this study can be made available by the corresponding author at reasonable request.

## Abbreviations

D&A: Disrespect and abuse
RMC: Respectful maternity care
PTSD: Post-traumatic stress disorder
GLM: General linear model
